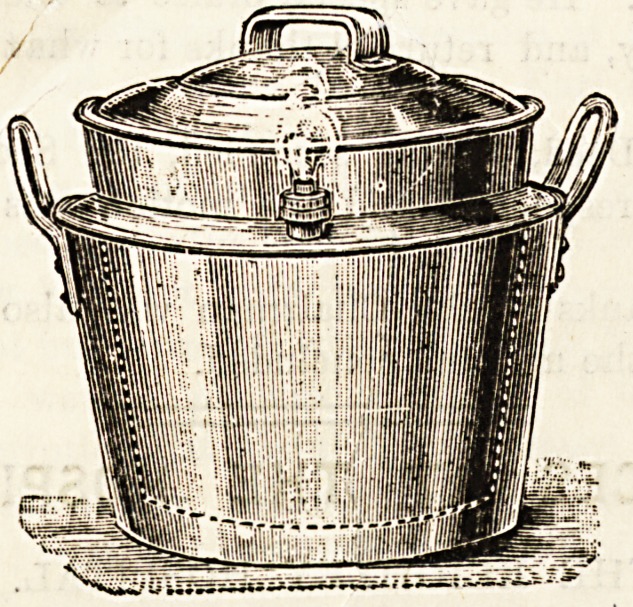# Practical Departments

**Published:** 1905-06-10

**Authors:** 


### PRACTICAL DEPARTMENTS.

### WELBANK'S BOILERETTE.

We have given this saucepan a careful practical test, and can sincerely accord it the warmest praise. It is an ideal cooker, thorough in its work, and most convenient and economical. For sick-room cookery it is invaluable; in fact, we hold that no sick-room should be without it, as it renders it possible to administer food at the moment it is required, which is so desirable. The inner receptacle, in which is placed the food, is encased in a jacket of steam. The safety-valve obviates all necessity for attention whilst the food is boiling, as neither boiling over nor burning can take place.

It should be introduced into the homes of the poor by the doctor or nurse, who will readily appreciate the chance it affords the invalid to secure properly cooked and unspoilt food. It would also prove a blessing to the country practitioner, who with its assistance might provide for a belated meal without the tedious waiting which he so frequently has to experience. But the uses and the advantages of the boilerette are too numerous to relate; they will be speedily apparent to those who make use of the excellent boilerette which is manufactured by Messrs. Welbank, Duplex Works, near Banbury.

## Figures and Tables

**Figure f1:**